# Double J stent malposition in the inferior vena cava: Two case reports and a literature review

**DOI:** 10.3389/fsurg.2022.910572

**Published:** 2022-10-21

**Authors:** Qihua Wang, Chen Shen, Yue Zhang, Lijie Wen, Bo Yang

**Affiliations:** Department of Urology, The Second Affiliated Hospital of Dalian Medical University, Dalian, Liaoning, China

**Keywords:** double j stent, malposition, intravascular migration, complication, treatment

## Abstract

Double J stent (DJS) malposition in the vascular system is a rare and severe complication. We present two cases in our department who went through DJS malposition in the inferior vena cava and our experience in treatment, and we reviewed the previous case reports to further summarize its underlying reasons and prevention and treatment methods.

## Introduction

Double J stent (DJS) placement is a common operation in the urology department to relieve or prevent ureteral obstruction and stricture. Although there are some common complications, such as hematuria, bladder irritation, flank, and suprapubic pain, or serious complications, such as vesicoureteral reflux, DJS migration, and DJS encrustation, it is generally a safe and effective procedure. In rare conditions, however, there are also cases where DJSs are misplaced or migrated to an abnormal position. We present two cases of DJS malposition in the inferior vena cava (IVC) and successful treatment in our center.

## Case presentation

### Case 1

**Figure 1:**
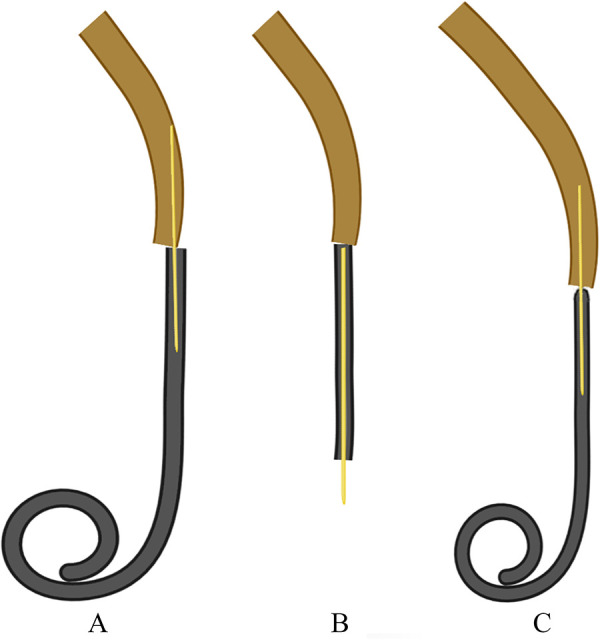
Pattern diagram of the properties of guidewire and double J stents (DJSs). (**A**) Inner diameter of the DJS is too large so the guidewire cannot fit the stent perfectly, which leads to DJS stuck at the ureteral orifice (arrow). (**B**) Another kind of a DJS with a blind proximal coil, which needs to be placed with a guidewire against its end. (**C**) DJS has a blunt-end design (arrow), and the inner diameter matches well with the guidewire, which can make the insertion safer and more accurate.

In 2006, a 54-year-old woman complained about right flank pain for 2 days, accompanied by a fever, as high as 39.1°C, for 1 day. Three months ago, she had undergone extracorporeal shockwave lithotripsy (SWL) for right kidney calculi, and scanty gravel was discharged. The physical examination showed obvious percussion pain in her right flank region. Blood and urine routines were normal. Ultrasound and plain CT revealed the diameter of the right renal pelvis was 4.5 cm and a ureteral calculus of 0.8 × 0.9 cm in the lower right ureter, about 3 cm away from the ureteral orifice. The patient’s temperature turned normal soon after antibiotic application. Therefore, ureteroscopic pneumatic lithotripsy was performed under epidural anesthesia without further contrast imaging such as retrograde pyelogram or on-table fluroscopy and ultrasound. The guidewire was reserved, and a double-opening F5 DJS was tried to be placed retrograde, but it was stuck at the ureteral orifice (Figure 1A). Instead, a DJS with a blind proximal coil was placed retrograde under a cystoscope, with a guidewire against its end (Figure 1B). There were no obvious postoperative complications such as hematuria, abdominal pain, fever, and the like. Unfortunately, no postoperative fluoroscopy and ultrasound were performed.

Two months later, the patient was advised to remove the DJS. As a long time had passed, the radiological data could not be obtained. According to the descriptions of the medical record system, routine laboratory tests including urinalysis were normal; fluoroscopy exhibited that the DJS was abnormally close to the spine, and plain CT revealed that the proximal end of the DJS was located in the IVC and the distal end penetrated and was attached to the distal ureter. After surgeons failed to find the distal end of the DJS in the bladder and collecting system in cystoscopy and ureteroscopy, an exploratory operation was performed using an extraperitoneal approach through an oblique incision of the right lower rectus abdominis muscle. The lower ureter, IVC, and external iliac vein were exposed, then the DJS was touched in the internal iliac vein. The distal end was located under the serosa of the distal ureter. The DJS was retracted completely without uncontrollable bleeding and thrombosis inside the stent. During a 1-year follow-up, the patient did not complain of any specific signs of hemorrhage, infection, or thrombotic complications, including fever, abnormal pain, anemia, and dyspnea. The semiannual ultrasound was clean.

### Case 2

In 2019, a 75-year-old woman with a history of postoperative recurrence of endometrial carcinoma was admitted to our hospital for chemotherapy. The physical examination showed mild percussion pain in her right flank region. Blood and urine routines were normal. A PET-CT showed the invaded right ureter, bladder, and adjacent intestine and the dilated right hydronephrosis ureter. After consulting with our urologist, a retrograde cystoscopic DJS placement was determined to be performed directly without further contrast imaging. Obvious resistance emerged when the hydrophilic guidewire went up about 10 cm. With an increased thrust, the guidewire went up smoothly after a sense of breakthrough; then, a DJS was placed along the guidewire without obvious postoperative complications. No on-table fluoroscopy or ultrasound was performed during the whole procedure.

**Figure 2:**
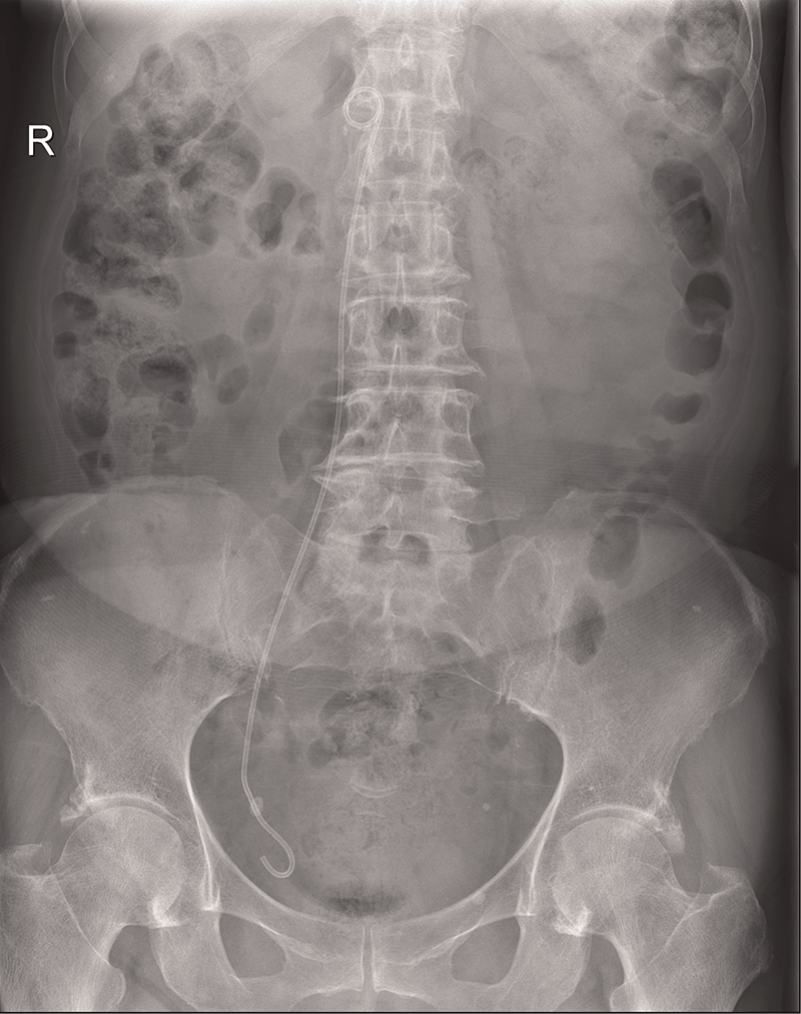
X-ray screening of a double J stent (DJS) malposition. The location of the DJS was abnormally closed to the spine.

**Figure 3:**
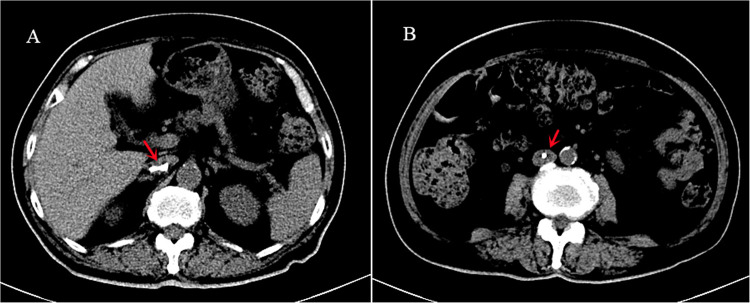
Computed tomography revealing the double J stent malposition (arrow). The proximal coil was at the level of upper renal polar.

After the procedure, the patient developed a fever, reaching 38.4°C. A blood routine showed mild elevated WBC, and urinalysis showed slight microscopic haematuria. An ultrasound revealed that her right hydronephrosis was the same as before, and there was no stent echo in her renal pelvis. An x-ray image hinted at a DJS malposition (Figure 2). A plain CT scan showed that the proximal end of the stent was located in the IVC at the level of upper renal polar (Figure 3). Approximately a week after the placement, the location of the distal coil was found to be intravesical under the cystoscope, and the DJS was slowly and integrally removed with retrieval forceps. After that, a percutaneous nephrostomy and catheterization were performed. The patient’s vital signs were stable all the time, and no hemorrhage symptoms such as tachycardia, unstable blood pressure, or abdominal pain occurred. After 8 months of follow-up, the patient is still on regular chemotherapy with normal renal function. The same as Case 1, the patient did not complain of specific signs; also, regular blood tests and ultrasound showed no abnormality.

## Discussion

Michalopoulos et al. (1) first described a DJS intravascular migration case in 2002. There are complications including flank pain, severe gross hematuria, infection, thrombosis, and so on. In the worst situation, the DJS may migrate to the cardiac and pulmonary vascular system following the blood cycle, rendering acute pulmonary embolism, tricuspid regurgitation, and endocarditis (1–4). To common sense, patients should tend to develop hematuria due to the connection between the vascular and urinary systems established by the DJS. However, surprisingly, many reports, including our cases, only disclosed microscopic or even no hematuria (2, 3, 5, 6) (Table 1). This situation is considered due to an obstructive clot inside the DJS (5). In addition, abnormal persistent flank, back pain, and high fever also hint at the existence of DJS malposition (4, 7–9) (Table 1). According to Tang’s (5) view, the intrapelvic pressure is naturally higher than that of the IVC (5–12 cm H_2_O), especially when there is obstructive hydronephrosis (even more than 22 cm H_2_O). Therefore, urine is more likely to reflux to blood rather than the opposite, which may lead to infection. In our cases, neither of them complained about abnormal pain and haematuria; only mild fever occurred in Case 2, which proved that the complication could be so covert. In addition, fever due to vesicoureteral reflux is easily seen after DJS placement. Our report reminds urologists of the possibility of urine reflux into the circulatory system directly.

**Table 1:** Previous case reports in the literature on intravascular migration of DJS.

Author and publication year	History and initial diagnosis	Reason and method of DJS placement	Symptoms	Location of the proximal (*P*) and distal (D) end	Duration of catheterization	Removal method	Thrombosis and anticoagulants use	Prognosis
Michalopoulos et al. 2002 (1)	Left kidney agenesis	Pyelolithotomy; OP, AG	Early postoperative pulmonary thromboembolism	*P*: right atrium	1 day	Endovascular	No thrombosis, postoperative prophylactic anticoagulants	NM
				D: left pulmonary arterial tree				
Ioannou et al. 2009 (3)	Left urolithiasis and recurrent pyelonephritis	To decongest a new episode of pyelonephritis; NM, RG	Microscopic hematuria after surgery	*P*: left common iliac vein	During hospitalization	OP	NM	Uneventful within 2.5 years of follow-up
				D: IVC				
Falahatkar et al. 2012 (11)	True urinary incontinence and left hydronephrosis after hysterectomy	To relieve urinary incontinence and hydronephrosis; US, RG	Gross hematuria	*P*: IVC next to the right atrial inlet	20 days	Endovascular	NM	Deep vein thrombosis developed 7 days after discharge, which needed specific treatment.
				D: left external iliac vein				
Tang et al. 2012 (5)	Right renal calculus	Post-PCNL; NM, AG	Asymptomatic	*P*: IVC	6 weeks	Endovascular	NM	
				D: right renal pelvis				
Sabnis et al. 2013 (13)	Right ureteric calculus	To relieve hydronephrosis and defer the lithotripsy; CS, RG	NM	*P*: right atrium	NM	OP	No thrombosis, postoperative prophylactic anticoagulants	Uneventful
				D: right external iliac vein				
Özveren et al. 2013	Right ureteral obstruction with history of chemo- and radiotherapy after radical hysterectomy	To relieve obstruction; CS, RG	Persistent gross hematuria	*P*: IVC up to the level of the hepatic veins	NM	CS	No	NM
				D: correct position				
Kim et al. 2014 (7)	Right ureteroneocystostomy with bladder rupture repair after a right lower ureteral transaction injury	To prevent urinary leakage; OP, RG	Mild back pain and suprapubic discomfort after voiding	*P*: right cardiac chamber	2 weeks	Endovascular	No	Normal urinary and vascular structures within 5 months of follow-up
				D: right ovarian vein and IVC				
Hastaoglu et al. 2014 (10)	Right ureteric calculus	Lithotripsy; NM, but not cystoscopic	Massive hematuria	*P*: right ventricle	3 years	Endoscopically and OP	No	Uneventful
				D: right ureteral lumen with calcification				
Farshi et al. 2015 (2)	Gestational right hydronephrosis and pyelonephritis	To relieve hydronephrosis; CS, RG	Asymptomatic	*P*: right ventricle	5 months	US	No	No adverse effects
				D: right ureteral lumen				
Hajji et al. 2015 (4)	Gestational hydronephrosis and pyelonephritis	To relieve hydronephrosis; NM	Palpitations and moderate right flank pain	*P*: right atrium	Placed in 12 weeks pregnant and removed after delivery	Endovascular	No	discharged on the following day
				D: right iliac vein				
Arab et al. 2016 (12)	Right ureteric calculus	Lithotripsy; US, RG	Gross hematuria	Completely in the pulmonary artery	2 weeks	Endovascular	Thrombus formation and prophylactic anticoagulant	No symptoms within 6 months of follow-up
Marques et al. 2018 (6)	Right renal lithiasis and pelvic stone	Lithotripsy for the pelvic stone; NM, RG	Asymptomatic	*P*: IVC	3 months	Endoscopically	No thrombosis, preoperative prophylactic anticoagulants	No long-term complications
				D: intravesical				
Tilborghs et al. 2019 (8)	Bilateral renal colic led by bilateral ureteric calculus	Relive symptoms before lithotripsy; NM, RG	Right renal colic persisted, gross hematuria, and high fever occurred	*P*: IVC at the level of the left renal vein	During hospitalization	CS	No	The laser lithotripsy was performed 4 weeks later, and the follow-up was uneventful
				D: intravesical				
Jiang et al. 2019 (9)	Left renal calculi	Pyelolithotomy; NM, RG	Persistent moderate flank pain	*P*: IVC at the level of the left renal vein	5 months	Percutaneous nephroscope under C-arm guidance	Small mural thrombus in the IVC, pre and postoperative anticoagulants	Uneventful
				D: left renal pelvic				

DJS, double J stent; US, ureterscopy; CS, cystoscopy, OP, open surgery; NM, not mentioned; RG, retrograde; AG, antegrade.

**Figure 4:**
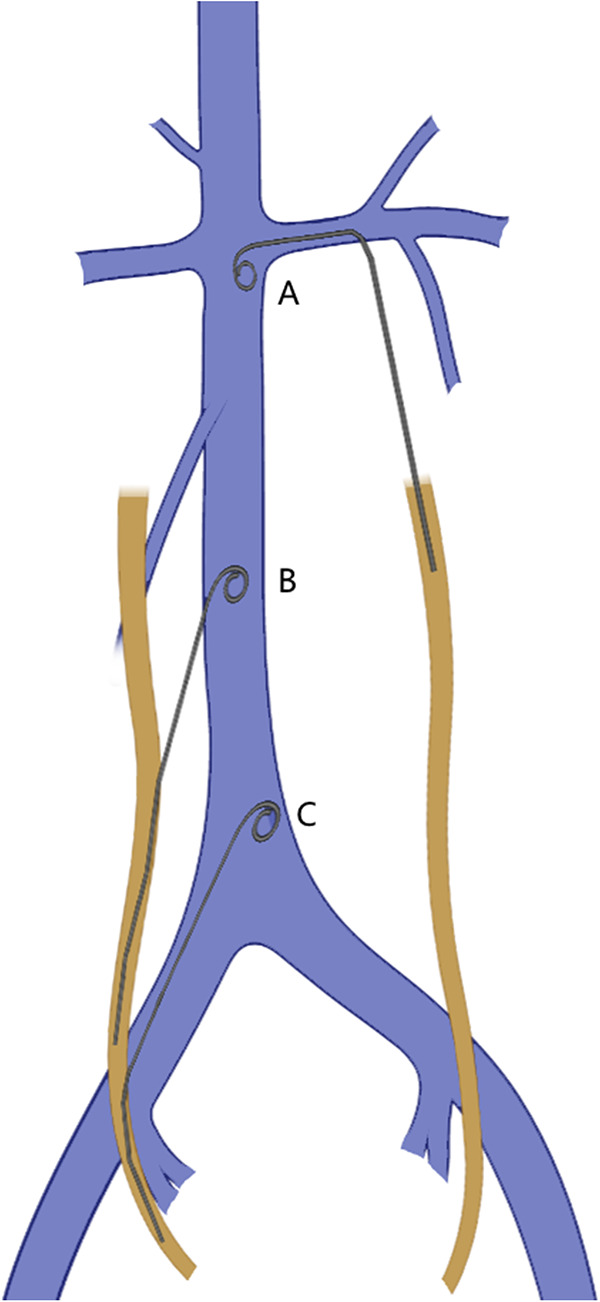
Common pathways for double J stents (DJSs) to enter the inferior vena cava (IVC). (**A**) through the left renal vein; (**B**) directly breaking into the IVC through the right ureter; (**C**) migration to the IVC via the right iliac vein.

In both of our cases, the DJSs were located in the IVC eventually. The reported locations of intravascular malposition of the DJS include the external iliac vein (3), IVC (6), ovarian vein (7), right ventricle (2, 10), right atrium (4), and pulmonary artery (1). For most situations, the malposition of DJS occurs on the right side (71.4% vs. 28.6%) (Table 1), the same as our facts that both cases occurred on the right side. The stent might break into the IVC through the right iliac vein or directly from the ureter; however, there were also cases that went through left disease (Figure 4) (9, 11). All postoperative x-ray examinations screened DJS malposition, which was aberrantly close to the spine. Our cases further proved this specific imagiological feature.

### Underlying reasons

We comprehensively analyze the underlying reasons, mainly related to the perforation of the ureter led by myriad factors (1): (1) Ureteral factors—Various kinds of damage to the ureter, such as calculus, chronic inflammation, malignant tumor, and pelvic surgery, may not only escalate its fragility but also cause ureteral stricture, which increases the resistance of DJS placement (8, 11). (2) The properties of guidewires and stents—If there is a mismatch between the guidewire and the stent, like the inner diameter of the DJS is too thick, it will not fit the guidewire perfectly, which will make it not amenable to insert and increase the risk of ureteral penetration. In addition, a blunt-end design can help to prevent penetration (Figure 1C). (3) Iatrogenic factors—Injury during lithotripsy and violent operation will lead to ureteral perforation. Following the standard steps of DJS placement is also of much importance, or the patient may develop unprecedented complications. In addition, an inappropriate pre- and intraoperative decision is also an important reason. A poor ureter condition is a common cause of failure in DJS placement, which requires a judicious termination of operation and then performing a nephrostomy instead. In addition, abrupt increases in intraabdominal pressure are also considered to be a factor in promoting the DJS shift (1). It should be noted that intraoperative fluoroscopy can timely reveal the DJS malposition.

In our cases, both the calculus in Case 1 and tumor infiltration in Case 2 could make the ureter more fragile and narrow. In addition, the DJS in Case 1 was not a blunt-end design and did not match the guidewire properly, which added much difficulty to the procedure. Moreover, there was obvious resistance before penetration. The absence of related knowledge and reckless operation against the resistance were notably important reasons in our cases.

### Prevention

To avoid this serious complication, physicians ought to evaluate the difficulty of insertion and the risk of ureteral perforation before surgery. Preoperative urography can provide a simple impression of ureteral conditions; however, to the best of our knowledge, the DJS can be placed successfully even if the urography presents a very narrow ureteral lumen. Ureteroscopic operation is recommended, especially when the resistance is abnormal during placement under a cystoscope, to reduce the risk caused by unidentified ureteral perforation. Unfortunately, there are reports of the DJS migrating to the IVC under ureteroscopic surgery, which was explained by the poor surgical view field (12, 13). Intraoperative C-arm fluoroscopy is optional to be performed if available and necessary. Postoperative x-ray is recommended, with which clinicians can carefully confirm the position of the stent. When an x-ray is not suitable, such as for pregnant women, an ultrasound as an alternative can safely confirm whether the guidewire and DJS are correctly located in the renal pelvis and bladder (2).

### Treatment

Regarding the treatment for DJS malposition, whether secondary thrombosis exerts a significant influence on decision-making needs to be confirmed. Therefore, contrast CT scanning should be performed when this complication is confirmed, which we did not notice and was risky. Tilborghs et al. (8) summarized that pharmacomechanical thrombolysis is indicated when vital signs are stable, there is no retroperitoneal hemorrhage or active bleeding focus, and adhesions are not present. Otherwise, urgent surgical thrombectomy should be considered. Before and after the surgical removal of the DJS, adequate prophylactic anticoagulation was recommended and adopted by many centers (1, 6, 9, 12, 13), although there is no consensus at present. In our cases, no prophylactic anticoagulant was prescribed, and patients did not present signs of thrombosis complications during follow-up. Considering the adverse effect of anticoagulants, we believe that the administration should be based on an individualized evaluation.

The removal method mainly depends on the position of the distal coil, the patient's general condition, and the available infrastructure (6). Removing an intravesical DJS endoscopically is feasible when there is no thrombosis and retroperitoneal hemorrhage. Previous reports (2, 6, 8) and our experience present that it is still a minimally invasive and safe choice when the surgical and anesthesia teams are fully prepared to cope with severe internal bleeding that may occur at any time. Tilborghs et al. (8) performed an immediate postoperative inferior venocavagraphy, which showed limited contrast leakage from the perforation site. On the opposite, emergency intervention or open surgery should be performed when the DJS completely enters the vascular system and cannot be found endoscopically. Open cardiothoracic surgery with cardiopulmonary bypass is crucial and the first choice, especially when patients have unstable vital signs and severe complications. Before surgery, it should be noticed that excessive encrustation is likely to form around the DJS. Extracorporeal shock wave lithotripsy or holmium laser can eliminate encrustation, which may fix the stent and make removing it difficult (10).

The advantages of our reports are that we provided two rare cases of DJS malposition in the IVC and tried to review every published report. Then, we summarized the underlying causes, prevention, and treatment methods, which we considered helpful to clinical practice. However, there are also a few limitations: We could only get information about Case 1 by retrieving the medical record because a long time has passed, so we could not provide any radiological evidence. Some references are outdated because DJS malposition is a rare complication and lacks authoritative guidelines.

## Conclusion

DJS placement is a common procedure in the urological department, but it should be noted that there is a possibility of ureteral perforation and even DJS malposition in the vascular system.

## Data Availability

The raw data supporting the conclusions of this article will be made available by the authors, without undue reservation.
